# An audit on admission clerkings across Lancashire and South Cumbria NHS Foundation Trust (LSCFT)

**DOI:** 10.1192/bjo.2021.842

**Published:** 2021-06-18

**Authors:** Sophie Edgell, Ahmed Sultan, Mohammed Hussain

**Affiliations:** 1 Chorley Mental Health Inpatient Unit; 2 The Harbour

## Abstract

**Aims:**

Following a Care Quality Commission (CQC) outcome showing that capacity assessments were not routinely completed on admission of patients we decided to complete an audit on current practice. We planned to review admission clerkings at Chorley Mental Health Inpatient Unit to assess quality, with the overall aim of putting measures in place to improve standards. We planned to make the results reflective of all psychiatry wards within Lancashire and South Cumbria NHS Foundation Trust (LSCFT) with the addition of a qualitative survey.

**Background:**

We are aware the standard of clerkings can vary and affect patient care. CQC outcome showed that that capacity assessment was not routinely documented and consultants have stated that clinical impressions are rarely documented in junior doctor clerkings. This audit allowed us to objectively assess these observations. We believed the results may show common themes throughout psychiatric practice more generally.

**Method:**

The gold standard was a 20 item list of expected components of a clerking, based on trust guidelines. A snapshot of current inpatients (n = 30) on 31/10/19 was taken. An Excel sheet was used for information gathering. Data were analysed and graphs created. A qualitative questionnaire on current practice was sent to trainees (n = 8) on different sites for an overview of practice across LSCFT. Therefore, a mixed-methods model was employed.

**Result:**

Items with the highest completion included clerking within 6 hours, face-to-face review with consultant completed within a week and current medication documentation. The items with the lowest completion included clinical impression documentation, bloods completed within 24 hours and documentation of capacity assessment and smoking/substances history. Common factors between clerkings with fewer completed items included poor patient engagement and patient transfer from another ward.

Qualitative survey (n = 8) showed that junior doctors across the health board are not using uniform methods for capacity documentation or an official checklist for clerking.

**Conclusion:**

We concluded that the low rate of capacity assessment completion was an important finding due to legal implications, and that there should be a uniform place for documentation of this. Physical health consequences of other missing components were explored. We will introduce standardisation of capacity assessment documentation and use of a clerking checklist, before re-auditing. The results were presented at local teaching and recommendations sent to Site Tutors for inclusion in local inductions.

